# Inhibitory effect of ginsenoside Rg3 combined with gemcitabine on angiogenesis and growth of lung cancer in mice

**DOI:** 10.1186/1471-2407-9-250

**Published:** 2009-07-23

**Authors:** Tai-Guo Liu, Ying Huang, Dan-Dan Cui, Xiao-Bing Huang, Shu-Hua Mao, Ling-Ling Ji, Hai-Bo Song, Cheng Yi

**Affiliations:** 1 Division of Abdominal Cancer, West China Hospital, Sichuan University, Chengdu 610041, Sichuan Province, PR China; 2 Department of Pathophysiology, West China School of Preclinical and Forensic Medicine, Sichuan University, Chengdu 610041, SichuanProvince, PR China; 3 Division of Hematology, Sichuan Province People's Hospital, Chengdu 610041, Sichuan Province, PR China; 4 Division of Ultrasonography, West China Hospital, Sichuan University, Chengdu 610041, Sichuan Province, PR China

## Abstract

**Background:**

Ginsenoside Rg3, a saponin extracted from ginseng, inhibits angiogenesis. The combination of low-dose chemotherapy and anti-angiogenic inhibitors suppresses growth of experimental tumors more effectively than conventional therapy or anti-angiogenic agent alone. The present study was designed to evaluate the efficacy of low-dose gemcitabine combined with ginsenoside Rg3 on angiogenesis and growth of established Lewis lung carcinoma in mice.

**Methods:**

C57L/6 mice implanted with Lewis lung carcinoma were randomized into the control, ginsenoside Rg3, gemcitabine and combination group. The quality of life and survival of mice were recorded. Tumor volume, inhibitive rate and necrosis rate were estimated. Necrosis of tumor and signals of blood flow as well as dynamic parameters of arterial blood flow in tumors such as peak systolic velocity (PSV) and resistive index (RI) were detected by color Doppler ultrasound. In addition, expression of vascular endothelial cell growth factor (VEGF) and CD31 were observed by immunohistochemstry, and microvessel density (MVD) of the tumor tissues was assessed by CD31 immunohistochemical analysis.

**Results:**

Quality of life of mice in the ginsenoside Rg3 and combination group were better than in the control and gemcitabine group. Combined therapy with ginsenoside Rg3 and gemcitabine not only enhanced efficacy on suppression of tumor growth and prolongation of the survival, but also increased necrosis rate of tumor significantly. In addition, the combination treatment could obviously decrease VEGF expression and MVD as well as signals of blood flow and PSV in tumors.

**Conclusion:**

Ginsenoside Rg3 combined with gemcitabine may significantly inhibit angiogenesis and growth of lung cancer and improve survival and quality of life of tumor-bearing mice. The combination of chemotherapy and anti-angiogenic drugs may be an innovative and promising therapeutic strategy in the experimental treatment of human lung cancer.

## Background

It is well established that the growth and progression of most solid cancers are angiogenesis – dependent; thus anti-angiogenic therapy is one of the most promising approaches for the treatment of cancers [1-3]. Folkman predicted that anti-angiogenesis would become the fourth treatment modality for cancer, besides surgery, chemotherapy, and radiation. More than 20 anti-angiogenic agents such as TNP-470, thalidomide, endostatin and angiostatin are subject to different phases of clinical trials [2]. Avastin (Bevacizumab) was approved by FDA in 2004 for the treatment of colorectal cancer [4]. Anti-angiogenic agents bear potential as a treatment of cancer.

Nowadays, efforts have been directed toward discovering of new anti-angiogenic agents. Ginsenoside Rg3, a saponin, extracted from ginseng, is a very powerful angiogenic inhibitor [2,5,6]. Some experiment findings indicate that ginsenoside Rg3 exhibits anticancer activity in vitro and in vivo models as a relatively safe medicine [5]. However, emerging data suggest that cancer therapy targeting only the tumor-existing vessels or tumor angiogenesis may not eradicate the tumor completely. Thus, the efficacy of anti-angiogenesis alone may be limited in advanced tumors [7].

Lung cancer is the leading cause of cancer death worldwide and gemcitabine is a nucleoside analog, accepted as a first-line chemotherapeutic agent for the disease. However, conventional chemotherapy schemes for the treatment of cancer mostly produce a limited improvement, associated with considerable side-effects and acquired drug resistance [5,8-10]. Results from animal models suggest that chronic administration of low doses of chemotherapy has an effect on the tumor and other compartments, mainly the vasculature [11].

In recent years there has been increasing interest in combining radiation or chemotherapy with angiogenesis inhibitors for tumor suppression – the combination may be more appropriate to produce improved efficacy and reduced toxicity by transcending each limitation [2]. Some results from animal models have suggested that the combination of low-dose chemotherapy with anti-angiogenesis therapy for solid tumors can suppress tumor growth more effectively than conventional chemotherapy or anti-angiogenic therapy alone [9,12-15]. However, the effectiveness of combination treatment of ginsenoside Rg3 and low-dose gemcitabine on lung cancer remains unclear. The present study was designed to evaluate the efficacy of ginsenoside Rg3 combined with low-dose gemcitabine on angiogenesis and growth of established Lewis lung carcinoma in mice.

## Methods

### Materials

A group of female C57BL/6 mice (6–8 week age) weighing between 18 g and 20 g were purchased from the Experimental Animal Center of Chinese Academy of Sciences. Mice were housed under pathogen-free conditions, and fed with animal chow and water ad libitum. Lewis lung carcinoma cell line was obtained from Cancer Research Institution of Sichuan University. Gemcitabine was supplied by Eli Lilly Company (USA). Ginsenoside Rg3 was extracted from northeast China's ginseng, and purity quotient was not less than 99.5%, and provided by YaTai Pharmaceutical Company (China). Mouse monoclonal antibody for vascular endothelial growth factor (VEGF) was purchased from Santa Cruz (USA). Mouse monoclonal antibody for CD31 and LSAB kit were purchased from Dako (Japan).

### Cell culture

Human Lewis lung carcinoma cells were cultured in Dulbecco's modified Eagle's medium (DMEM) supplemented with 10% fetal bovine serum plus ampicillin and streptomycin routinely, and incubated in 5% CO_2 _at 37°C.

### Design of animal experiments

All animal procedures were approved by the Animal Care and Scientific Committee of Sichuan University. The tumor tissues from Lewis lung carcinoma mice were triturated and prepared into cell suspensions (dilution 1:5 with normal saline). The cells harvested from xenografts were adjusted to a concentration of 1×10^7^/ml, and 0.2 ml cell suspensions were injected subcutaneously into the armpit of right anterior superior limbs of female C57BL/6 mice. Seven days later, when the tumors were palpable, the mice were randomized into the following four groups (10 mice per group): a gemcitabine group injected intraperitoneally with gemcitabine on every 3rd day (10 mg/kg) in a total of 6 treatments; a ginsenoside Rg3 group, which received ginsenoside Rg3 by gastric perfusion daily (20 mg/kg) for 18 consecutive days; a combination group (gemcitabine plus ginsenoside Rg3) treated with gemcitabine and ginsenoside Rg3 on the same schedule as above, and a control group injected with normal saline subcutaneously. All treatments lasted for 18 days.

### Tumor growth, side effects and quality of life of mice

The length and width of tumor were callipered every 4 days for tumor growth, and tumor volume (TV) was estimated using the formula: TV (mm^3^) = (width^2 ^× length)/2. During the experiment period, side effects such as weight loss, change in behavior and feeding, reaction to stimulation, ruffling of fur and psychosis (distress) were observed. When a mouse died, the size of tumor and the number of living days were recorded.

### Inhibitive rate of tumor

18 days after the treatment, the remaining mice in all groups underwent color Doppler before they were sacrificed and then the size and weight of tumors were recorded. Inhibitive rate of tumor was calculated using the formula [16]: inhibitive rate of tumor (%) = (1- average tumor weight in treated group/average tumor weight in control) × 100%.

### Necrosis rate of tumor

Necrosis rate of tumor was assessed using pathological slices way [8,17]. Necrosis rate of tumor was determined using the following formula: necrosis rate of tumor = tumor necrosis area/whole tumor area × 100% (tumor necrosis area = the largest diameter × the smallest diameter of the tumor necrosis area; whole tumor area = the largest diameter × the smallest diameter of the tumor area)

### Observation of necrosis of tumor

The size, shape of tumor, ultrasonic echo from tumor inner to known liquefied tumor and necrotic tissues were detected under the two-dimensional ultrasound by color Doppler (ACUSON1228ST) with 5 MHz frequency.

### Detection of dynamic parameters of arterial blood flow intumor by color Doppler flow imaging (CDFI)

Signals of blood flow in tumor were evaluated by CDFI with color Doppler at 5 MHz. Briefly, the tumor was carefully scanned form different angles with attention to vascularity and intratumoral and peritumoral arterial flow signals. Vascularity could be arterial or venous. When the margins of the tumor were clearly seen, the location of the largest arterial vessel relative to the tumor was determined. Pulsed Doppler sample volume was located on the largest arterial vessel and the angle of the transducer was adjusted manually to obtain the maximum amplitude and frequency shift (Doppler angle < 60°). Arterial blood stream frequency spectrum was captured and stored for later analysis of parameters.

Signals of blood flow in tumor of CDFI were classified into four grades based on the criteria of Adler [18]: 0, no blood flow signals detected within the tumor; I, minimal blood flow (one or two dot-like or a thin- and short-like blood flow signals detected within the tumor); II, moderate blood flow (up to three dot-like blood flow signals or one longer blood flow signals detected within the tumor); and III, abundant blood flow (more than five dot-like blood flow signals or two longer blood flow signals detected within the tumor).

Artery parameters in tumor such as peak systolic velocity (PSV, cm/s), end diastolic velocity (EDV, cm/s) and resistive index (RI, RI = PSV-EDV/PSV) were evaluated by blood stream frequency spectrum. A mean value for each parameter of intratumoral and peritumoral arterial blood flow was obtained from at least three different measurements.

### Immunohistochemical detection of CD31 and VEGF

Tumor tissues were fixed immediately in 10% buffered formalin phosphate and embedded in paraffin. Immunohistochemical staining was performed using a labeled streptavidin-biotin method. Briefly, the dewaxed, rehydrated sections (5 μm) were treated with 0.3% hydrogen peroxide in methanol for 30 min to block endogenous peroxidase activity and then washed three times with 0.01 M phosphate buffered saline (PBS, pH 7.2). The sections were permeabilized with ethylene diamine tetraacetic acid (EDTA) buffer solution (pH 9.0) for 15 min with microwave. Nonspecific binding was blocked with 2% normal goat serum at room temperature for 15 min followed by treatment with primary antibody at 37°C for 2 h (anti-CD31 diluted for 1:100 and anti-VEGF diluted for 1:50). After washed by PBS, sections were then incubated with biotin labeled secondary antibody diluted for 1:100 at 37°C for 30 min and washed with PBS. Peroxidase conjugated streptavidin was added for 20 min and then washed with PBS. Finally, sections were developed with 3, 3-diaminobenzidine and hydrogen peroxide and counterstained with hematoxylin. For the negative control, PBS was used instead of primary antibody. The sections were analyzed by light microscopy.

VEGF staining was considered positive if unequivocal yellow brown staining was seen in the tumor cell cytoplasm, and the immunoreactivity was scored semiquantitatively as the intensity of the immunoreactive reaction and positive percent of tumor cells [16]. The intensity of the immunoreactive reaction were graded as 0, no immunoreactivity; 1, weak intensity; 2, moderate intensity; 3, strong intensity. Positive percent were graded as 0 to 4 score (0, < 10%; 1, 10%–24%; 2, 25%–49%; 3, 50%–75%; 4, ≥ 75%). After adding the scores of intensity and positive percent, we rescaled to score 0 to 1 as negative (-), 2 to 3 as weak expression (1+), 3 to 4 as moderate expression (2+) and above 5 as strong expression (3+).

### Detection of microvessel density (MVD)

MVD was assessed by immunohistochemical analysis with antibodies to the endothelial marker CD31 and determined according to the method of Weidner and colleagues [19]. Briefly, the immunostained sections were initially screened at low magnifications (40× and 100×) to identify hot spots, which are the areas of highest neovascularization. Any yellow brown stained endothelial cell or endothelial cell cluster that was clearly separate from adjacent microvessels, tumor cells, and other connective tissue elements was considered a single, countable microvessel. Within the hot spot area, the stained microvessels were counted in a single high-power (200×) field, and the average vessel count in 3 hot spots was considered the value of MVD. All counts were performed by three investigators in a blinded manner. Microvessel counts were compared between the observers and discrepant results were reassessed. The consensus was used as the final score for analysis.

### Statistical analysis

TV, necrosis rate of tumor, MVD, PSV and RI were analyzed by one-way analysis of variance (ANOVA), followed by the Student's *t *test. Data of inhibitive rate of tumor was analyzed by Chi-square test. Survival curves were constructed according to the Kaplan-Meier method and statistical significance was determined by the log-rank test. VEGF and grade of CDFI were analyzed by Kruskal-Wallis test. All statistical analyses were performed using SPSS 11.5 software package. All P values were two-sided and P < 0.05 was considered as the significant level of difference.

## Results

### Tumor volume, inhibitive rate and survival rate

**Figure 1:**
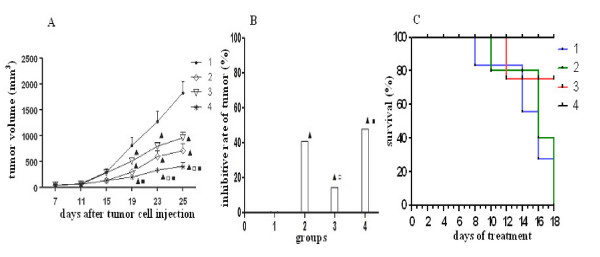
**Effect of ginsenoside Rg3 and gemcitabine on tumor growth and survival**. A: tumor growth curve; B: inhibitive rate of tumor; C: survival curve. Tumor volume (TV) (mm^3^) = (width^2 ^× length)/2. Inhibitive rate of tumor (%) = (1- average tumor weight in treated group/average tumor weight in control) × 100%. Survival curves were constructed according to the Kaplan-Meier method and statistical significance was determined by the log-rank test. 1: control group; 2: gemcitabine group; 3: ginsenoside Rg3 group; 4: combination group. ^▲^P < 0.05 vs control group; ^□^P < 0.05 vs gemcitabine group; ^■^P < 0.05 vs ginsenoside Rg3 group. Combined therapy with ginsenoside Rg3 and gemcitabine enhanced efficacy on suppression of tumor growth and prolongation of the survival.

The treatment began on the 7th day after the mice were transplanted with tumor cells. Treatment with gemcitabine or ginsenoside Rg3 alone showed an appreciable decrease in tumor volume compared with that of the controls during treatment period. Remarkably, the combination group showed enhanced efficacy on tumor volume suppression (Figure 1A). In addition, inhibitive rate of tumor in the combination group was significantly higher than that of the ginsenoside Rg3 group. Inhibitive rate of tumor in the combination group also was higher than that in the gemcitabine group, but the difference did not reach statistical significance (P > 0.05) (Figure 1B).

18 days after the treatments, the number of living mice in the control, gemcitabine, ginsenoside Rg3 and combination group were 6, 7, 9 and 10, respectively. The mice died from tumor deterioration and side effects of therapy, excluding improper experimental manipulation by mice anatomy. The cumulative survival rate was 60%, 70%, 90% and 100%, respectively. Combined therapy with ginsenoside Rg3 and gemcitabine resulted in prolonged survival compared with the control or gemcitabine group (P < 0.05) (Figure 1C).

### Tumor necrosis and necrosis rate of tumor

**Figure 2:**
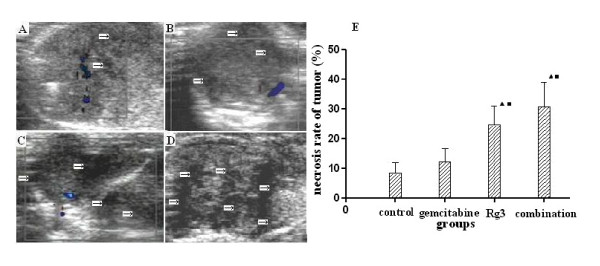
**Effect of ginsenoside Rg3 and gemcitabine on necrosis of tumor**. Multi-local and larger areas of tumor necrosis (arrow) in the combination (Figure 2D) and ginsenoside Rg3 group (Figure 2C), and only smaller areas of tumor necrosis detected in the gemcitabine (Figure 2B) and control group (Figure 2A) detected by color Doppler ultrasound. E: necrosis rate of tumor (necrosis rate of tumor = tumor necrosis area/whole tumor area × 100%). Necrosis rate of tumor was assessed using pathological slices way. ^▲^P < 0.05 vs control group; ^■^P < 0.05 vs gemcitabine group.

Tumor necrosis was detected by color Doppler before the mouse was killed. As shown in Figure 2, there were obviously multi-local and larger areas of tumor necrosis in the combination (Figure 2D) and ginsenoside Rg3 group (Figure 2C), and only smaller areas of tumor necrosis detected in the gemcitabine (Figure 2B) and control group (Figure 2A). The necrosis rate of tumor in both the combination and the ginsenoside Rg3 group was evidently higher than that in the gemcitabine and control group (P < 0.05). The necrosis rate in the combination group also was higher than that in the ginsenoside Rg3 group (30.7% vs 24.5%), however, without significant difference (P > 0.05) (Figure 2E).

### Signals of blood flow in tumor

The number of arterial blood flow frequency spectrum detected in the control, gemcitabine, ginsenoside Rg3 and combination group were 6, 5, 7 and 6, respectively. Detection rates of arterial flow in the control, gemcitabine, ginsenoside Rg3 and combination group were 100% (6/6), 71.4% (5/7), 77.8% (7/9) and 60% (6/10), respectively.

**Figure 3:**
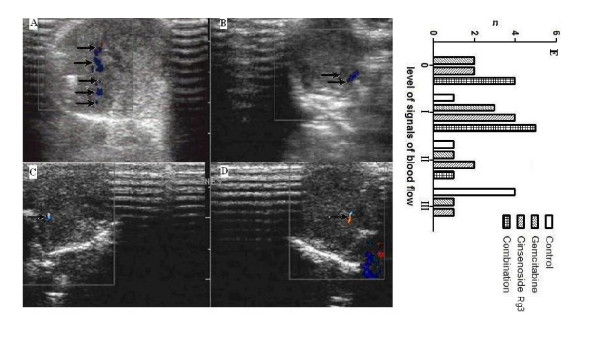
**Observation of blood flow in tumors by color Doppler flow imaging (CDFI)**. A: more than five dot-like blood flow signals and three club-like blood flow signals (arrow) detected in tumors in the control group; B: dot- and club-like blood flow (arrow) in tumors in the gemcitabine group; C: a short-like blood flow (arrow) in tumors in the ginsenoside Rg3 group; D: a thin- and short-like blood flow (arrow) in tumors in the combination group. E: levels of signals of blood flow. Signals of blood flow in tumors of CDFI were classified into four grades: 0, no blood flow signals detected within the tumor; I, minimal blood flow (one or two dot-like or a thin- and short-like blood flow signals detected within the tumor); II, moderate blood flow (up to three dot-like blood flow signals or one longer blood flow signals detected within the tumor); and III, abundant blood flow (more than five dot-like blood flow signals or two longer blood flow signals detected within the tumor).

As shown in Figure 3, color Doppler flow imaging showed that signals of blood flow in tumor were the worst in the combination group and the best in the control group. The signals of blood flow were mainly level 0-I (9/10) in the combination group, while mostly at level III (4/6) or level II (1/6) in the control group. The signals were intermediate in the gemcitabine and ginsenoside Rg3 group.

### Dynamic parameters of arterial blood flow in tumor

**Figure 4:**
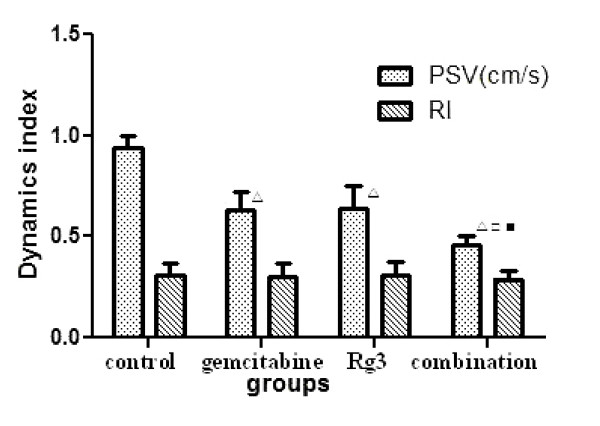
**Changes of dynamic parameters of arterial blood flow in tumors by color Doppler flow imaging**. PSV: peak systolic velocity; RI: resistive index, RI = PSV-EDV/PSV (EDV: end diastolic velocity). ^▫^P < 0.05 vs control group; ^□^P < 0.05 vs gemcitabine group; ^■^P < 0.05 vs ginsenoside Rg3 group.

PSV in tumor in the combination group decreased significantly compared with the other three groups (P < 0.05). No statistically significant changes in RI were found among groups (P > 0.05) (Figure 4).

### MVD and VEGF expression

**Figure 5:**
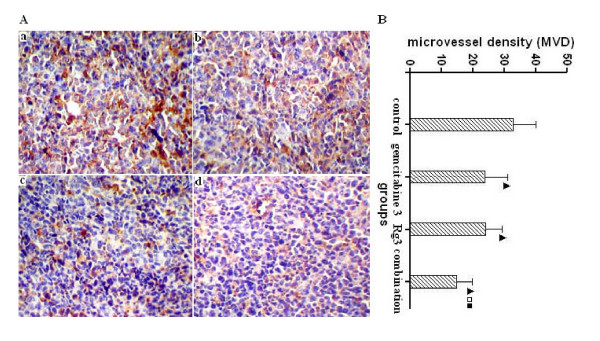
**Immunohistochemical staining of CD31 and microvessel density (MVD)**. A: CD31 (200×). The blood vessels in tumor tissues were stained in yellow brown. a: control group; b: gemcitabine group; c: ginsenoside Rg3 group; d: combination group. B: MVD. MVD was determined by counting the number of microvessels per high-power field (hpf) in the section with an antibody reactive to CD31. ^▫^P < 0.05 vs control group; ^□^P < 0.05 vs gemcitabine group; ^■^P < 0.05 vs ginsenoside Rg3 group.

**Figure 6:**
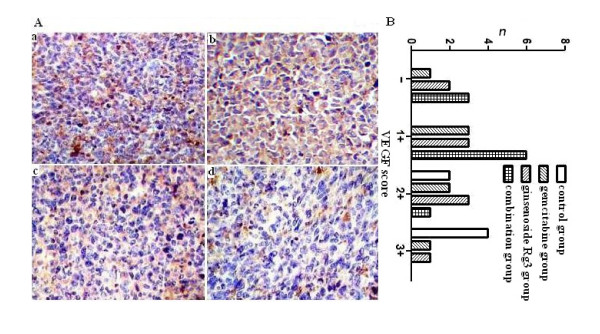
**Effect of ginsenoside Rg3 and gemcitabine on VEGF expression of tumors**. A: Immunohistochemical staining of VEGF (200×). a: control group; b: gemcitabine group; c: ginsenoside Rg3 group; d: combination group. B: VEGF score. VEGF staining was scored semiquantitatively as the intensity of the immunoreactive reaction and positive percent of tumor cells. Score 0 to 1 was as negative (-), 2 to 3 as weak expression (1+), 3 to 4 as moderate expression (2+) and above 5 as strong expression (3+). 1: control group; 2: gemcitabine group; 3: ginsenoside Rg3 group; 4: combination group.

MVD was determined by counting the number of the microvessels per high-power field (hpf) in the section with an antibody reactive to CD31 (Figure 5A). Compared with the control group, MVD value in the ginsenoside Rg3, gemcitabine and combination group decreased obviously, especially in the combination group (P < 0.05) (Figure 5B). There was positive expression of VEGF in the cytoplasm of some tumor cells. The VEGF expression in the ginsenoside Rg3, gemcitabine and combination group was lower than that in the control group (P < 0.05), and VEGF expression in the combination group was lower than in the gemcitabine and ginsenoside Rg3 group (P < 0.05) (Figure 6). The results indicated that ginsenoside Rg3 inhibited tumor angiogenesis and its anti-angiogenic effect was further improved when combined with gemcitabine.

### Side effects and quality of life of mice

There were no significant abnormalities in psychosis, status of activity, reaction to stimulation, loss of weight, appetite or depilation of mice in the ginsenoside Rg3 group though it was not the case in the combination, control or gemcitabine group. The quality of life of mice in the ginsenoside Rg3 group was the best and the worst was found in the gemcitabine group. The side effects were reduced and quality of life improved in the combination group compared with the gemcitabine group. The findings indicated that ginsenoside Rg3 might potently decrease side effects of therapy and improve quality of life of tumor-bearing mice.

## Discussion

Anti-angiogenic therapy for cancer is aimed to produce a 'dormant' state in which tumor cell proliferation and tumor expansion are stalled by inhibiting tumor-related angiogenesis, thus depriving the tumors of essential nutrients and oxygen [20]. Anti-angiogenic therapy has shown both protective and therapeutic anti-tumor effects. However, the fact that angiogenesis inhibitors do not act directly on the tumor cells suggests that complete eradication of a tumor is unlikely with the anti-angiogenic treatment alone [8,10].

Gemcitabine, 2-2-difluoro-2-deoxycytidine (dFdC), is one of the most effective chemotherapy medicaments against lung cancer. dFdC is a deoxycytidine analog that inhibits DNA synthesis. After taken up by cells, dFdC is phosphorylated to its active metabolites, which inhibits DNA chain elongation, leading to DNA fragmentation and cell death [8,10,21]. In addition, dFdC is also capable of inhibiting ribonucleotide reductase, an enzyme with a key role in DNA repair procedures [7,22]. Chemotherapy is one of the mainstays for cancer therapy. However, drug resistance and relatively strong toxic side effects (sometimes even unbearable) complicate treatment [8]. Thus, it is conceivable that anti-angiogenic combined with chemotherapy may exert greater effect on tumor growth, even eliminating tumor.

At present, anti-cancer drugs are moving toward natural chemical compounds from animals and plants. Given advances in Chinese herbal medicine, researchers are becoming increasingly interested in detecting anti-tumor components in Chinese herbal medicine. Ginsenoside Rg3 is an effective chemical trace component extracted from ginseng with C_42_H_72_O_13 _framework and 784 Da molecular weight [5]. Ginsenoside Rg3 has been shown to exhibit anti-cancer activity, and the anti-tumor effect has been attributed to the actions of inhibiting growth, invasion, metastasis of various tumors and neovascularization [2,5].

In the present study, gemcitabine combined with ginsenoside Rg3 showed a significant tumor growth inhibition than ginsenoside Rg3 alone. The data suggest that the combination of low-dose chemotherapy with anti-angiogenic therapy for lung cancer can suppress tumor growth more effectively than anti-angiogenic therapy alone. Furthermore, the inhibitive rate of tumor of the combination therapy group was higher than gemcitabine group without statistical significance. It seems that the effect of anti-tumor growth in the combination group is not superior to chemotherapy alone. However, data from color Doppler show that combination therapy led to multi-local, larger areas of tumor necrosis, while only smaller areas of tumor necrosis presented in the gemcitabine group. The necrosis rate of tumor in both the combination and ginsenoside Rg3 group was evidently higher than that in the gemcitabine group. The findings suggest that ginsenoside Rg3 or combined with gemcitabine can promote tumor necrosis more effectively than chemotherapy alone. Morioka et al. reported that in the therapy of mouse chondrosarcoma, combination therapy of angiogenic inhibitor PRP-B and chemotherapeutic drug ET-743 caused significantlygreater necrosis relative to any individual treatment, although tumor volume measurements did not parallel the necrosis values, who considered the curative effect in the combination group to be superior to the single drug group [8].

Tumor volume and necrosis rate of tumor are usually used to evaluate treatment effect of chemotherapy. In fact, tumor volume may actually increase with increasing tumor necrosis [8,23]. Thus, estimating the tumor volume alone is no longer an adequate parameter when evaluating therapy [24]. Some researchers consider that tumor necrosis rather than tumor volume is used as a criterion for determining treatment effect of chemotherapeutic agents [8,25].

Anti-angiogenic therapy is initially active on capillary formation. Subsequent hypoxia induces tissue degeneration and tumor cell eradication [24]. The higher necrosis rate of tumor observed with ginsenoside Rg3 treatment alone and combination treatment as compared with gemcitabine treatment alone may be due to the effect of ginsenoside Rg3. Ginsenoside Rg3, an angiogenic inhibitor, restrains tumor-dependent angiogenesis and then aggravates ischemia and hypoxemia of tumor tissues.

Angiogenesis plays an important role in both tumor growth and metastasis [26]. Angiogenesis is tightly regulated by pro-angiogenic and anti-endothelial growth factors. VEGF is one of the most essential pro-angiogenic growth factors [27], and it also appears to be critical in the angiogenic process [28]. MVD is accepted as a standard indicator of angiogenesis [24] and VEGF expression is strictly correlated with MVD [29,30]. In the present study, MVD in the ginsenoside Rg3, gemcitabine and combination group decreased obviously, especially in the combination group. In addition, VEGF protein expression in tumors was also consistent with that of MVD, which were in agreement with the findings of Xu TM, et al [6,31,32]. Our data indicated that ginsenoside Rg3 and gemcitabine reinforced each other's inhibitory effect of angiogenesis by decreasing MVD value and VEGF expression.

Doppler sonography is noninvasive, easy to perform, which can give important information about tumor vessels [24] and the hemodynamic characteristics of tumors [24,31,32] and monitor the responses to anti-tumor therapy [31]. In the present study, color Doppler flow imaging showed that signals of blood flow and PSV in the combination group decreased significantly compared with the other three groups.

Altogether, data above indicate that ginsenoside Rg3 and low-dose gemcitabine may potentiate each other's antitumor activities. The mechanism responsible for the interaction between gemcitabine and low-dose chemotherapy remains unclear. On one hand, the mechanism for the anti-tumor effect of ginsenoside Rg3 is associated with inducing apoptosis, regulating cell cycle, blocking angiogenesis, and inhibiting metastasis [5,10]. On the other hand, being a cytotoxic agent, gemcitabine could interfere with DNA synthesis and induce DNA breakage, thus causing tumor cell apoptosis [10,33-35]. It has been reported that gemcitabine could result in downregulation of tumor-cell-produced VEGF by inducing tumor cell apoptosis [10,36]. In addition, some results of recent experimental studies have suggested that frequent administration of certain cytotoxic agents at low doses increases the anti-angiogenic activity of the drugs [9,10,37]. The above effects may contribute to the synergistic inhibition of tumor angiogenesis and growth. However, the exact mechanisms need further study.

Ginseng is one of the most popular herbal medicines and has been used to proactively promote health, vitality, and longevity in Asian countries for more than 2000 years [2,38,39]. In recent years, ginseng has gained significant popularity in western societies [5,40], and has been included in the Pharmacopoeias of several western countries such as Germany, France, Austria, and the United Kingdom [38,41]. Many studies have reported that ginseng promotes a wide range of pharmacologic activities in the immune, cardiovascular, endocrine, and central nervous systems [38,42,43]. Several clinical trials have demonstrated that ginseng could improve overall quality of life in healthy volunteers or patients with certain diseases, such as diabetes [38,41]. Recently, a large, population-based cohort study (1,455 breast cancer patients) showed that ginseng use after cancer diagnosis, particularly current use, was positively associated with quality of life scores, with the strongest effect in the psychological and social well-being domains [38].

Ginsenosides are the major active components of ginseng, which have been shown to have a variety of beneficial effects, including immunomodulatory [2], antioxidant [44], anti-stress [2], anti-inflammatory [44], anti-aging activities [2], and anti-fatigue [2], et al. In addition, ginsenoside Rg3 is a relatively safe and effective medicine [5]. Some results demonstrated that ginsenoside Rg3 not only had no side effects on marrow, heart, lung, liver, kidney, and the nervous system [5], but also could improve the living quality of mice with tumor [6,30]. In this study, ginsenoside Rg3 combined with gemcitabine demonstrated enhanced efficacy on the prolongation of survival. In addition, quality of life of mice in the ginsenoside Rg3 and combination group were better than in the control and gemcitabine group. To date, the exact mechanism of ginsenoside Rg3 improving quality of life remains unclear. We believe that the effects may be attributed to its wide spectrum of medicinal effects, which endow ginsenoside Rg3's special predominance differing from other angiogenic inhibitors. In 2000, Rg3 appeared in the market as a new anti-cancer drug called "Shen-Yi Capsule" in China. Now, Ginsenoside Rg3 has been applied into clinical therapy as a Class I new drug in China [2].

## Conclusion

The present study suggests that ginsenoside Rg3 combined with gemcitabine may significantly inhibit angiogenesis and growth of lung cancer and improve survival and quality of life of tumor-bearing mice. The combination of chemotherapy and anti-angiogenic drugs may be an innovative and promising therapeutic strategy for the experimental treatment of human lung cancer.

## Abbreviations

VEGF: vascular endothelial growth factor; TV: tumor volume; MVD: microvessel density; CDFI: color Doppler flow imaging; PSV: peak systolic velocity; RI: resistive index; EDV: end diastolic velocity.

## Competing interests

The authors declare that they have no competing interests.

## Authors' contributions

TGL, YH, DDC, XBH and CY designed and performed the experiments, and contributed to manuscript writing. XBH performed pathology experiments. HBS performed ultrasound experiments. SHM and LLJ analyzed the data. All authors read and approved the final manuscript.

## Pre-publication history

The pre-publication history for this paper can be accessed here:

http://www.biomedcentral.com/1471-2407/9/250/prepub
